# Giant Rashba effect at the topological surface of PrGe revealing antiferromagnetic spintronics

**DOI:** 10.1038/s41598-017-02401-z

**Published:** 2017-06-23

**Authors:** Soma Banik, Pranab Kumar Das, Azzedine Bendounan, Ivana Vobornik, A. Arya, Nathan Beaulieu, Jun Fujii, A. Thamizhavel, P. U. Sastry, A. K. Sinha, D. M. Phase, S. K. Deb

**Affiliations:** 1 Synchrotrons Utilization Section, Raja Ramanna Centre for Advanced Technology, Indore, 452013 India; 2 Department of Condensed Matter Physics and Materials Science, Tata Institute of Fundamental Research, Homi Bhabha Road, Colaba, Mumbai, 400005 India; 3 Synchrotron SOLEIL, L′Orme des Merisiers, Saint-Aubin, BP 48, FR-91192 Gif-sur-Yvette Cedex, France; 4 Istituto Officina dei Materiali (IOM)-CNR, Laboratorio TASC, in Area Science Park, S.S.14, Km 163.5, I-34149 Trieste, Italy; 5 Materials Science Division, Bhabha Atomic Research Centre, Mumbai, 400085 India; 6 Solid State Physics Division, Bhabha Atomic Research Centre, Mumbai, 400085 India; 7 UGC-DAE Consortium for Scientific Research, Khandwa Road, Indore, 452001 India; 8 International Centre for Theoretical Physics, Strada Costiera 11, 34100 Trieste, Italy; 9 Indian Institute of Technology Bombay, Powai, Mumbai, 400076 India

## Abstract

Rashba spin-orbit splitting in the magnetic materials opens up a new perspective in the field of spintronics. Here, we report a giant Rashba spin-orbit splitting on the PrGe [010] surface in the paramagnetic phase with Rashba coefficient *α*_*R*_ = 5 eV*Å*. We find that *α*_R_ can be tuned in this system as a function of temperature at different magnetic phases. Rashba type spin polarized surface states originates due to the strong hybridization between Pr 4*f* states with the conduction electrons. Significant changes observed in the spin polarized surface states across the magnetic transitions are due to the competition between Dzyaloshinsky-Moriya interaction and exchange interaction present in this system. Presence of Dzyaloshinsky-Moriya interaction on the topological surface give rise to Saddle point singularity which leads to electron-like and hole-like Rashba spin split bands in the $\bar{{\boldsymbol{Z}}}^{\prime} -\bar{{\boldsymbol{\Gamma }}}-\bar{{\boldsymbol{Z}}}$ and $\bar{{\boldsymbol{X}}}^{\prime} -\bar{{\boldsymbol{\Gamma }}}-\bar{{\boldsymbol{X}}}$ directions, respectively. Supporting evidences of Dzyaloshinsky-Moriya interaction have been obtained as anisotropic magnetoresistance with respect to field direction and first-order type hysteresis in the X-ray diffraction measurements. A giant negative magnetoresistance of 43% in the antiferromagnetic phase and tunable Rashba parameter with temperature makes this material a suitable candidate for application in the antiferromagnetic spintronic devices.

## Introduction

One of the main challenges in the field of spintronics is to manipulate the spin structures by electric and spin currents. In this context the materials with Rashba spin-orbit (SO) coupling^1^ have gained a huge amount of interest due to their use in the Spintronics technology^2, 3^. Rashba effect in the non-magnetic systems has been extensively studied^4–7^ but there are very few reports on the magnetic metallic systems showing the Rashba effect^8–10^. In ferromagnetic (FM) materials with the SO splitting the magnetization reversal gives rise to Rashba effect which can be used for the giant magnetoresistance devices and spin transfer torque device applications^9, 10^. Antiferromagnetic (AFM) materials are the hidden magnets and the magnetism in these systems is difficult to probe experimentally. The magnetization reversal in the AFM systems will not make any change because of zero net magnetic moment. In such a case, the rotation of the spins will lead to the change in the resistance and has important technological applications^11, 12^. The anisotropic magnetoresistance in these AFM systems is linked to the SO interaction. Hence, the AFM systems which have significant anisotropy can be the promising candidates for the AFM spintronic devices^13^.

**Figure 1:**
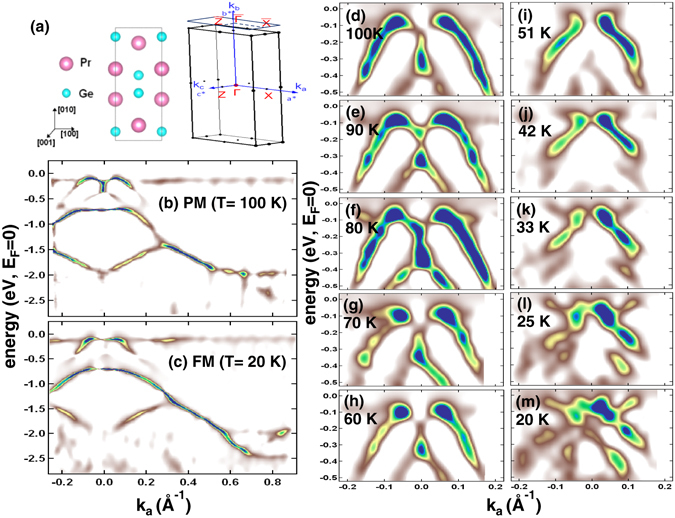
(**a**) Crystal structure of bulk PrGe and Brillouin zone with the high-symmetry points. The projected surface 2D Brillouin zone is also shown. Temperature dependent band structure of PrGe [010] surface at the (**b**) PM phase at 100 K and (**c**) FM phase at 20 K showing the Rashba spin split bands near the Γ point. In both the PM and FM phase a flat band below *E*_*F*_ is observed (discussed in text). Detail band structure study at different temperatures is performed to understand the nature of the spin split bands across the magnetic transitions. Rashba SO split bands are shown in a zoomed scale around the Γ point at (**d**) 100 K, (**e**) 90 K, (**f**) 80 K, (**g**) 70 K, (**h**) 60 K, (**i**) 51 K, (**j**) 42 K, (**k**) 33 K, (**l**) 25 K and (**m**) 20 K.

In recent years there has been flurry of research in understanding the anisotropic magnetic properties of several Pr-based compounds^14–18^. Pr-based compounds exhibit a variety of ground state properties due to the critical role of crystalline-electric-field effects, quadrupolar fluctuations *etc*.^14–18^. The mechanism of the heavy Fermion behavior in Pr-compounds is quite different from the usual Kondo route to heavy Fermions in Ce-based compounds. The crystal structural, transport and magnetic properties of PrGe single crystal have been studied in great detail^19–21^. PrGe crystallizes in CrB type crystal structure with *Cmcm* space group (Fig. 1(a))^19^. Magnetic studies on PrGe single crystal showed two magnetic transitions at 44 K and 41.5 K related to the AFM and FM transitions respectively^19^. Interestingly, a higher effective magnetic moment of Pr in PrGe has been observed for [010] crystallographic orientation ~3.90 *μ*_*B*_, which is not only higher than the effective moment of free Pr^3+^ ion ~3.58 *μ*_*B*_ but also higher than the moment observed along [100] (~3.78 *μ*_*B*_) and [001] (~3.71 *μ*_*B*_) crystallographic orientations. Since, the effective magnetic moment is related to the magnetocrystalline anisotropy (MCA), which is intrinsically linked to the SO interaction. Hence, to understand the origin of different magnetic ground states in PrGe it is utmost important to investigate the nature of SO interaction present in this system.

In the present work, we have investigated the band structure and electronic density of states by angle resolved photoemission (ARPES) and resonant photoemission measurements, respectively. We report that the Rashba type SO splitting observed on the topological surface of PrGe [010] single crystal is due to the fact that the system behaves like a weak ferromagnetic system which is associated with the spin-canting antiferromagnetic coupling between the Pr atoms. The magnetism is due to the indirect exchange interaction between the localized moment carried by the Pr 4*f* electrons which induces a spin-polarization in the conduction electrons and give rise to Rashba type spin split bands. There is a competition between Dzyaloshinsky-Moriya (DM) interaction^22^ and exchange interaction which actually changes the spin-polarization of the conduction electrons and give rise to different magnetic phases in PrGe.

## Results and Discussions

High resolution ARPES measurements have been performed to determine the electronic band structure of PrGe [010] single crystal at *hν* = 28 eV. The surface 2D Brillouin zone which has been probed is shown in Fig. 1(a). In Fig. 1(b,c) we have shown the band structure in the paramagnetic (PM) phase at 100 K and FM phase at 20 K, respectively. The most interesting observation in the band structure is that a flat band at −0.12 eV shows a Rashba type SO splitting in the PM and FM phase (Fig. 1(b,c)) near the Γ point. The spin split bands have hole-like character. The presence of Rashba SO splitting indicates that there is symmetry breaking at the surface which leads to spin-polarized surface states. We find that the spin polarized surface states which lies between −0.5 eV to *E*_*F*_ gets modified across the magnetic transition (Fig. 1(b,c)). A non-dispersive flat band at −0.12 eV emerges either due to the singular density of states within the dislocations^23^ or due to the interaction between electrons or holes on the surface of materials because of nontrivial topology of the electronic spectrum in bulk. In the gapless topological systems, it is reported that the bulk-surface and bulk-vortex correspondence produces such flat bands on the surface of the system or in the core of topological defects^23–25^. We find that the Rashba effect observed in this magnetic system is quite different from the Rashba effect reported for the other magnetic systems like Gd, Tb *etc*.^8, 9^, hence its origin need to be explored.

To understand the role of surface states and its correlation with the magnetism present in this system, we have performed a detailed band structure study as a function of temperature as shown in Fig. 1(d–m). The behavior of the split bands in the PM phase remains similar (Fig. 1(d–h)) between 100 K to 60 K. A drastic change is observed in the bands at and below 51 K (Fig. 1(i)) which is close to the AFM transition. In the AFM phase, the spin split feature in the hole-like bands disappears and is found to be overlapped with the electron-like bands. On further lowering the temperature, in the FM phase, we have observed both the overlapped electron-like and hole-like bands with prominent spin split features (Fig. 1(k–m)). The changes in the band structure across the magnetic transition is due to the mixing of the Pr 4*f* states with the other valence states in this system which actually causes a change in the spin polarization of the conduction electrons. Similar kind of changes in the band structure have been observed in CeSb across the PM to AFM phase transition^26^ which was attributed to the mixing of Sb 5*p* bands with the Ce 4*f*  bands.

**Figure 2:**
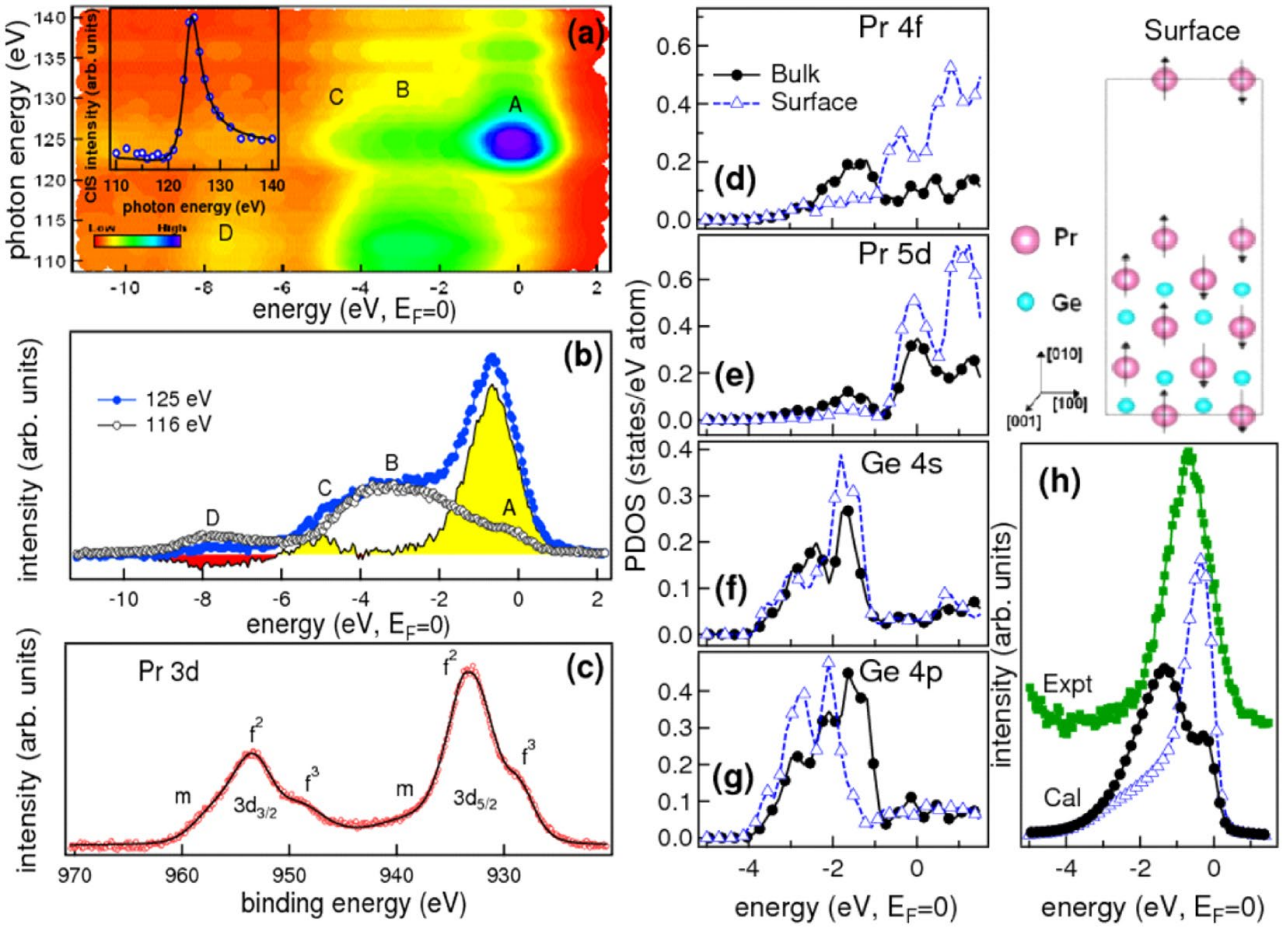
Comparison of the experimental VB with the theoretical DOS calculations: (**a**) RPES data across the Pr 4*d*–4*f* resonance are shown in a contour plot. The inset shows the CIS spectrum for feature A, where the open circle is the experimental data and solid line is the fitted Fano profile. (**b**) The on-resonance spectrum at h*ν* = 125 eV and off-resonance spectrum at h*ν* = 116 eV along with the difference spectrum. The yellow and red shaded regions in the difference spectrum corresponds to the Pr 4*f* states and the other valence states respectively. (**c**) Pr 3*d* core level with the observed features *f* ^2^,  *f* ^3^ and *m* marked in the spectrum. In (**d**–**g**) we have shown the PDOS of Pr 4*f*, Pr 5*d*, Ge 4*s* and Ge 4*p* states calculated using GGA method for bulk (black filled circle) and surface slab with Pr terminated surface (blue open triangle). The crystal structure of the surface slab with the anti-parallel orientation of spins of Pr atoms is shown. (**h**) experimental partial density of Pr 4*f* state (as in (**b**), shown by green filled square) compared with the partial density of Pr 4*f* states from bulk (black filled circle) and the surface slab (blue open triangle) calculations.

We find that the Rashba type SO splitting is large in the PM phase. Hence, to understand the character of the bands which give rise to the splitting, we have performed the resonant photoemission (RPES) measurements at room temperature. RPES has been performed across the Pr 4*d*–4*f* resonance in the photon energy range from 110 to 140 eV. The RPES data are plotted in the contour plot as shown in Fig. 2(a), where 4 prominent features marked as A, B, C and D at −0.65, −3.2, −5 and −8 eV respectively, are observed. Across the Pr 4*d* to 4*f* resonance, feature *A* shows a significant enhancement in intensity (Fig. 2(a)). The constant initial state (CIS) spectrum for the feature *A* at −0.65 eV has been plotted in the inset of Fig. 2(a) using the standard method discussed elsewhere^27, 28^. To understand the character of this feature Fano line profile^29^ of the form $\sigma (h\nu )={\sigma }_{a}\frac{{(q+\varepsilon )}^{2}}{1+{\varepsilon }^{2}}+{\sigma }_{b}$ and *ε* = (*hν* − *E*_0_)/*W* has been fitted and shown in the inset of Fig. 2(a) with a solid line. Here, the parameters *E*_0_, *q* and *W* represents the resonance energy, discrete/continuum mixing strength and the half-width of the line, respectively. The value of the parameters determined from fitting are *E*_0_ = 125 ± 0.02 eV, *q* = 3.69 ± 0.01 and *W* = 1.92 ± 0.01 eV. The larger value of *q* indicates that the states at −0.65 eV BE (feature A) is localized in nature. Two broad features B and C do not show the resonance. We find that the Pr 4*d*–4*f* resonance in PrGe is quite different from the results reported for thick Pr films^30^. The localized Pr 4*f* states at −3.6 eV in bulk Pr films show a larger enhancement than the features near *E*_*F*_ which are mainly the Pr 5*d* states^30^. However, in PrGe the enhancement of the states near E_*F*_ gives a clear indication that both the localized and the itinerant character of the Pr 4*f* states play important role in the magnetism of this system. Similar enhancement of Pr 4*f* states near E_*F*_ has been observed in the RPES spectra of high *T*_*C*_ superconductor Y_1−*x*_Pr_*x*_Ba_2_Cu_3_O_7−*δ*_^31^ which has been attributed to the hybridization of the Pr 4*f* states with the Cu 3*d* and O 2*p* states in this system. For PrGe, the resonance has been observed at ~125 eV which is much above the Pr 4*d* threshold energy (114 eV) which confirms that there is a finite hybrization present in this system. *E*_0_ (~125 eV) obtained for PrGe matches well with the reported value of the *E*_0_ for thick Pr films^30^ and high *T*_*C*_ superconductor Y_1−*x*_Pr_*x*_Ba_2_Cu_3_O_7−*δ*_^31^.

The signature of hybridization between the 4*f* states and the conduction electrons is well known to give rise to features in the core level spectrum and is studied in detail in other rare-earth based systems^27, 32^. Pr 3*d* core level spectrum is shown in Fig. 2(c) where SO splitting of 20.1 eV has been obtained between the Pr 3*d*_5/2_ and 3*d*_3/2_ peaks. Extra features marked as  *f* ^3^ and *m* in Fig. 2(c) have been observed for both the Pr 3*d*_5/2_ and 3*d*_3/2_ peaks. The asymmetric feature *m* arises due to the multiplet electronic states and has also been seen in other Pr based systems^33^. The main peak  *f* ^2^ is associated with the poorly screened 3*d* ^9^ *f* ^2^ states while the other feature marked as  *f* ^3^ arises from the 3*d* ^9^ *f* ^3^ configuration^34, 35^.  *f* ^3^ feature has been attributed to 5*d* → 4*f* satellite^34, 35^ which also confirms the hybridization of the Pr 4*f* states with the conduction electrons. Since Pr 4*f* states carries the local moment hence the strong hybridization of the Pr 4*f* states with the conduction electron leads to the spin polarization which give rise to the Rashba effect in this system.

It is reported that for the light rare-earth elements, which has less than the half the maximum number of 4*f* electrons exhibit only weak magnetism^36^. This weak magnetism is associated with the antiferromagnetism and arises due to the spin canting present in the system. In this case, the magnetic moments which occupy each site of a regular lattice are assumed to be equivalent but not exactly antiparallel, leaving a net magnetization on the system. This net magnetization in the canted antiparallel arrangement gives rise to the exchange splitting. This kind of antisymmetric interaction which causes the spin canting is known as DM interaction and the microscopic mechanism is related to the spin-orbit coupling. The competition between the DM interaction and the isotropic ferromagnetic exchange interaction gives rise to the magnetism in the weak ferromagnetic system.

To explore the above mentioned possibilities in PrGe, we have performed the first principles density of states (DOS) calculations of [010] surface of PrGe within DFT using GGA method by considering the experimental lattice parameters^19^ and compared it with the bulk calculations as shown in Fig. 2(d–h). The SO coupling for the Pr 4*f* states have been included in the calculations. For the surface calculations, we have used the periodic supercell or slab model approach^37^. The vacuum layers of thickness of 10 *Å* were added to the surface to minimize interatomic interactions between periodic images of the slabs. The slab was periodically repeated in three dimensions to facilitate calculations in reciprocal space. For our calculations, we have selected Pr-terminated PrGe [010] surface (see Fig. 2) in the anti-parallel spin configuration (for Pr atoms) incorporating SO coupling. All the atoms in the surface slab were fully relaxed. Our relaxed positions of atoms indicated deviation of less than ±0.001 *Å* from the initial positions. We have shown the PDOS of Pr 4*f* and Pr 5*d* states of the top Pr atoms in the surface slab calculation in Fig. 2(d,e). Compared to the bulk PDOS, we find that the Pr 4*f* and the Pr 5*d* states has a larger intensity at and near *E*_*F*_ (Fig. 2(d,e)) in the surface slab calculation. The energy position of the PDOS of all the states remain same in both the bulk and the surface slab calculation except for the Ge 4*p* state, which show a 0.46 eV shift towards higher energy for the surface calculation as compared to the bulk calculation (Fig. 2(g)). The Ge 4*s* PDOS (Fig. 2(f)) shows a slightly higher intensity in the surface calculation. The broadened PDOS of Pr 4*f* states obtained from the surface slab calculation (Fig. 2(h)) shows a good agreement with the experimental PDOS obtained from the difference of the on-resonance and the off-resonance spectra at 125 eV and 116 eV as shown in Fig. 2(b). There is a small shift of about 0.3 eV between the peak position of the experimental PDOS of Pr 4*f* states with that from the surface slab calculation of Pr 4*f*  PDOS. The difference between the theoretical and the experimental DOS is because the calculation has been performed for the ideal sample and the sample related effects like the complex magnetism present in this system, the actual condition of the spin polarization and antisite disorders or defects present in the system are not taken into account. At and near *E*_*F*_, the density of states shows a strong hybridization of the Pr 4*f* states with the Pr 5*d* states. We find that the total magnetic moment obtained for the top Pr atom in the surface slab calculation is very small about 0.009 *μ*_*B*_ which further supports the argument that the PrGe system is indeed similar to weak ferromagnetic systems.

**Figure 3:**
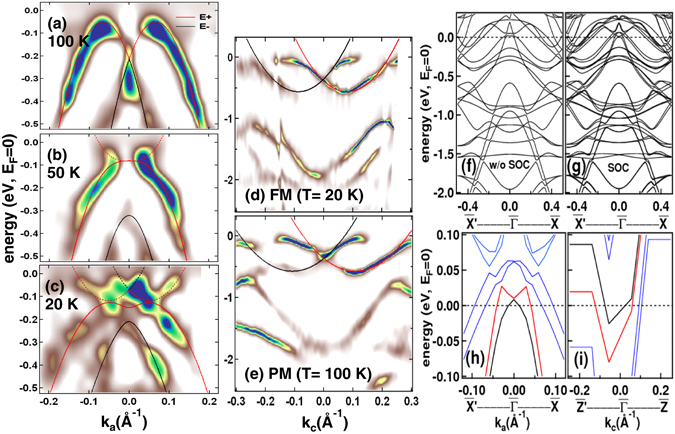
Experimental band structure compared with the simulated energy dispersion curves and the theoretical band structure calculations: (**a**–**c**) shows the experimental band structure at the 100 K (PM phase), 50 K (near AFM phase) and 20 K (FM phase) respectively, compared with the simulated energy dispersion curves in the *k*_*a*_ direction. The simulation has been carried out with the equation described in the text. Solid and dotted lines represents hole-like and electron-like bands, respectively. E+ (red line) and E− (black line) corresponds to the bands simulated with spin index +1 and −1, respectively. (**d**,**e**) shows the experimental band structure at 20 K (FM phase) and 100 K (PM phase) compared with the simulated energy dispersion curves in the *k*_*c*_ direction. The theoretical band structure of the Pr terminated [010] surface with the anti-parallel spin orientation showing the bands (**f**) without considering the spin-orbit coupling and (**g**) with the spin-orbit coupling in the *k*_*a*_ direction. (**h**) shows zoomed region of the theoretical band structure calculation along the *k*_*a*_ ($\bar{X}^{\prime}$-$\bar{{\rm{\Gamma }}}$-$\bar{X}$) direction and (**i**) shows the zoomed region of theoretical band structure calculation along the *k*_*c*_ ($\bar{Z}^{\prime}$-$\bar{{\rm{\Gamma }}}$-$\bar{Z}$) direction.

To further understand the behavior of the energy dispersion curves at different magnetic phases due to the DM interaction, we have shown the band structure in both *k*_*a*_ and *k*_*c*_ direction in Fig. 3(a–e), respectively. Interestingly, different hole-like and electron-like Rashba spin-split bands are observed in the *k*_*a*_ and *k*_*c*_ directions. The appearance of electron-like and hole-like bands in different direction of k-space is related to the presence of Saddle point van Hove singularity in the system^38^. Saddle point singularity is a clear signature of the spontaneous symmetry breaking due to the DM interaction and has been reported for the gapless topological systems with chiral magnetic structures^24, 25^. In presence of DM interaction^39^ the single particle energy for the magnetic system is: ${E}_{k,\sigma }={\textstyle \tfrac{{\hslash }^{2}}{2{m}^{\ast }}}[{({k}_{x}-\sigma {k}_{0}\sin \theta )}^{2}+{k}_{y}^{2}]-{E}_{R}\,{\sin }^{2}\,\theta -\sigma {[{({J}_{0}S)}^{2}+{\alpha }_{R}^{2}({k}_{x}^{2}{\cos }^{2}\theta +{k}_{y}^{2})]}^{1/2}$. Here *k*_*x*_ and *k*_*y*_ are the momentum parallel to the surface (*XY*-plane) which is similar as the *k*_*c*_ and *k*_*a*_ direction respectively in the present scenario. *σ* is the carrier spin index = ±1, *θ* denotes the angle of the magnetization with respect to the *Z*-axis (perpendicular to the surface), m* is the effective mass and *J*_0_ *S* = Δ represents the coupling between the magnetic impurity and the carriers where *J*_0_ is the exchange strength and *S* is the spin of the magnetic impurity. *α*_*R*_ denotes the coupling constant in the SO Hamiltonian which is described by $\tfrac{{\hslash }^{2}{k}_{0}}{{m}^{\ast }}$. Rashba energy *E*_*R*_, is described by $\tfrac{{m}^{\ast }{\alpha }_{R}^{2}}{2{\hslash }^{2}}$. We have simulated the energy dispersion curves considering three major conditions for the DM interaction in this system: 1) magnetization axis (*M*) is exactly perpendicular to the surface, in this case *θ* = 0, 2) *M* is along *Z*-axis but tilted in *XY*-plane and 3) *M* is parallel to the *Y*-axis and the direction of moment tilt away from the *Y*-axis in the direction perpendicular to surface, in this case *θ* = 90°. The detail discussion on the simulation is presented in the supplementary information.

In the experimental band structure (Fig. 1(d–m)) it is quite clear that the shift of the bands in the momentum space from the Γ point is almost constant however there is a drastic changes in the shape of the bands across the magnetic phase transition. Hence, in the simulation as shown by the solid lines in Fig. 3(a–c) we have varied the value of *k*_0_, m*, Δ and *θ*¸ to understand their actual effect on the band structure. We find that the experimental observations matches very well with the third condition where *M* is parallel to *Y*-axis (supplementary information Fig. 2(c)). The important result is that only in the case of magnetization axis being parallel to *Y*-axis (*θ* = 90°) (supplementary information Fig. 3(g–i)) the Rashba SO split bands lie well below *E*_*F*_. Hence, the physical effect of *θ* is it causes a shift of the valence band maximum. From the analysis performed on the experimental band structure, we have obtain the value of *k*_0_ ≈ 0.11 ± 0.005 *Å* ^−1^ along the *k*_*c*_ direction which is same in both PM and FM phase. The value of *k*_0_ in PrGe [010] is about 7.3 times higher than the value of *k*_0_ ≈ 0.015 *Å* ^−1^ as reported for Gd [0001]^8, 9^. The electron-like states in the *k*_*c*_ direction is lowered by an energy *E*_*R*_ ≈ 0.58 ± 0.002 eV *w*.*r*.*t*. *E*_*F*_. The value of m* is negative for the hole-like bands and positive for the electron-like bands. The important result obtained along the *k*_*a*_ direction for the hole like bands is that the effective mass m* is found to increase in the FM phase (0.466 ± 0.005 *m*_*e*_) and in the AFM phase (0.326 ± 0.005 *m*_*e*_) than in the PM phase (0.176 ± 0.005 *m*_*e*_). In the *k*_*c*_ direction the effective mass of the electron-like states is found to be two times less as compared to the hole-like states observed in the *k*_*a*_ direction for that particular phase. The value of *α*_*R*_ obtained is ≈5 ± 0.02 eV*Å* at 100 K. This is probably the largest value of *α*_*R*_ obtained so far for the metallic surface states. We find that the value of *α*_*R*_ decreases across the phase transitions from ~2.7 ± 0.02 eV*Å* in near AFM phase to ~1.89 ± 0.02 eV*Å* in the FM phase. The highest value of *α*_*R*_ in the paramagnetic phase over a wide temperature range from 300 K to 60 K makes it an important material for the spintronics applications. We also report that the Rashba parameter *α*_*R*_ in PrGe can be tuned by varying the temperature. Δ mainly causes a shift in the spin-up and the spin-down bands in the near AFM phase and in the FM phase which is quite evident in Fig. 3(b,c). Highest value of Δ ≈ 0.120 eV is obtained for the AFM phase (50 K, Fig. 3(b)) which reduces to ≈0.030 eV in the FM phase (20 K Fig. 3(c)). Spin canting with respect to the *Y*-axis (*k*_*a*_ direction) can be determined from the value of *α*_*R*_ and Δ such that the canting angle *δ*(*k*_*a*_) = tan^−1^(*α*_*R*_(*k*_*a*_)/Δ). The value of canting angle is almost 90° ± 0.02° in the PM phase, 87.5° ± 0.02° in the near AFM phase and 89.1° ± 0.02° in the FM phase. Different spin canting angle at near AFM and the FM phase and finite interaction between the hole-like and the electron-like states clearly indicates that there is change in the spin-polarization of the conduction electrons at different magnetic phases. The simulated curves in Fig. 3(d,e) show poor matching with the experimental bands above the saddle point near *E*_*F*_ the reason could be due to the coulomb interaction between the electron-like and hole-like states in the vicinity of the Fermi-level which has not been taken into account in the simulation. Broken space inversion symmetry has been reported to give rise to giant Rashba effect in the non-magnetic systems like BiTeI (*α*_*R*_ ~ 3.8 eV*Å*)^40^ and Bi/Ag(111) surface alloy (*α*_*R*_ ~ 3.05 eV*Å*)^7^. Giant Rashba effect in PrGe (*α*_*R*_ ~ 5 eV*Å*) is also due to broken inversion symmetry under the influence of DM interaction.

We have performed the theoretical band structure for the surface slab calculation to further understand the behavior of the bands in both the *k*_*a*_ and *k*_*c*_ direction. The top Pr atom carries very small magnetic moment (0.009 *μ*_*B*_) and are perpendicular to [010] surface of PrGe (Fig. 2) which is similar to the condition obtained by simulation. The effect of SO coupling (SOC) on the band structure calculation is shown in Fig. 3(f,g) respectively. The calculations done without incorporating SOC clearly does not reveal any Rashba splitting of bands which, in turn, imparts conviction to the presence of Rashba splitting observed in the calculated band structure incorporating SOC. The zoomed region around $\bar{{\rm{\Gamma }}}$ point in Fig. 3(h,i) clearly show the existence of saddle point singularity in this system and the orbital switching from hole-like to electron-like orbital in the $\bar{X}^{\prime}$-$\bar{{\rm{\Gamma }}}$-$\bar{X}$ and $\bar{Z}^{\prime}$-$\bar{{\rm{\Gamma }}}$-$\bar{Z}$ directions, respectively for the SOC calculations. The asymmetric bands in the $\bar{Z}^{\prime}$-$\bar{{\rm{\Gamma }}}$-$\bar{Z}$ direction is a clear signature of the SOC and the effect of magnetic anisotropy present in this system. Some differences between the theoretical and experimental band structure could be due to the sample related effects as well as the experimental conditions which could not be taken into account in the surface slab calculations.

**Figure 4:**
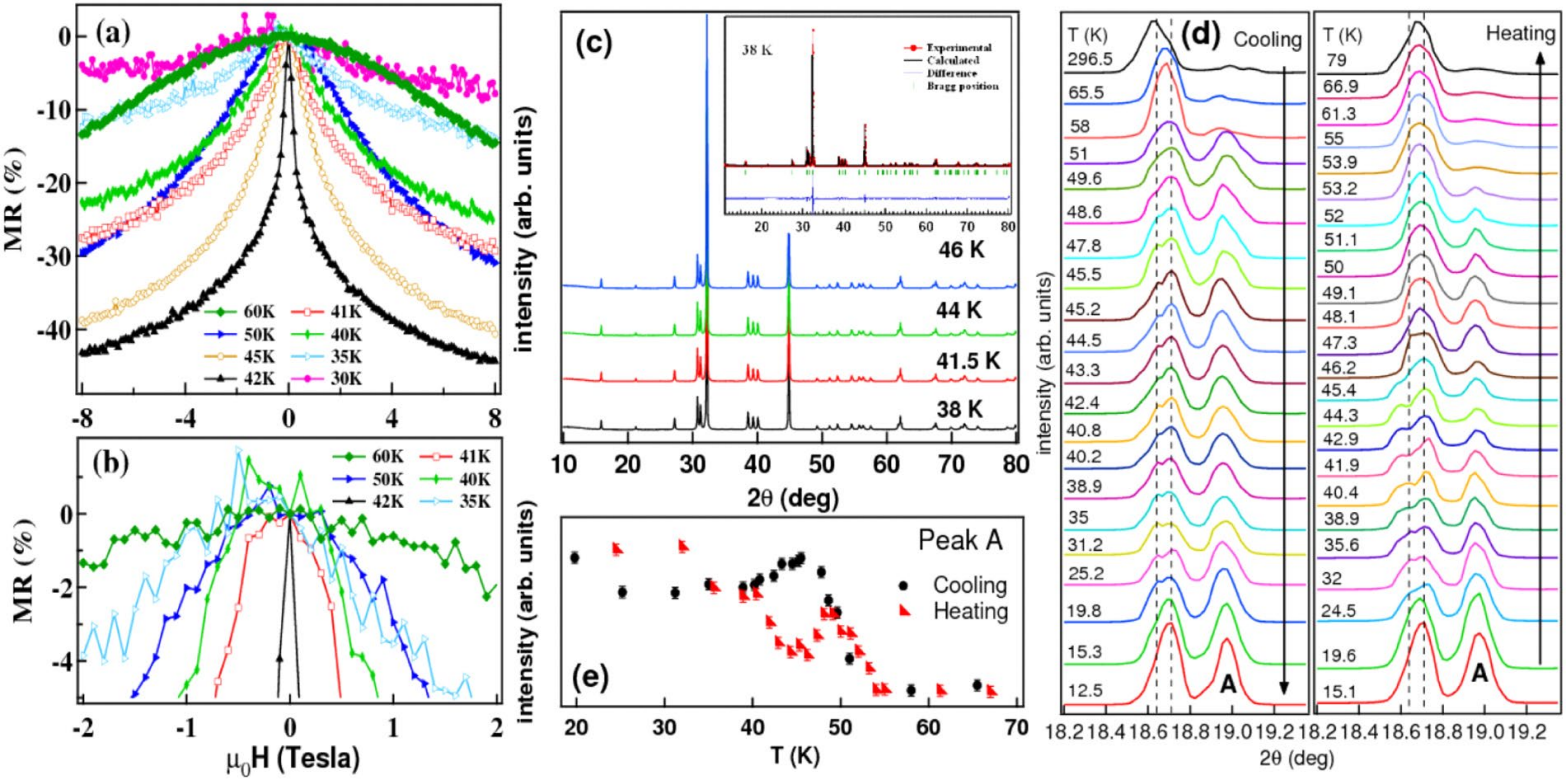
Temperature dependent MR and XRD measurements on PrGe: (**a**) Field dependence of the MR along the [010] direction performed at different temperatures. The MR is quantified in percentage by the following relation: MR(T, H) = ${\textstyle \tfrac{[\rho (T,H)-\rho (T,H=0)]}{(\rho (T,H=0))}}\times 100{\rm{ \% }}$. (**b**) Zoomed MR in the low field region to show the anisotropic MR behavior *w*.*r*.*t*. field direction. Temperature dependent XRD patterns measured with (**c**) Cu K_*α*_ laboratory based source and (**d**) linearly polarized synchrotron source at 13 KeV energy. Inset in (**c**) shows the rietveld refinement of the X-ray diffraction pattern measured with Cu K_*α*_ source at 38 K confirming the CrB type crystal structure with Cmcm space group. (**e**) The intensity of the peak *A* marked in (**d**) is plotted as a function of temperature which shows a clear hysteresis in both PM to AFM and AFM to FM phase transitions. The hysterisis in XRD pattern is characteristic of the first order nature of the magnetic transition.

For further understanding the spin structures present in this system, we have performed the magnetoresistance (MR) measurements. MR as a function of temperature is shown in Fig. 4(a,b). Negative MR has been observed in the PM phase at 60 K (Fig. 4(a)), which clearly indicates that there is a net magnetization present in this system. A large MR of 43% at 8 T field is observed in the AFM phase at 42 K. However in the FM phase 30 K the MR is more positive than the AFM phase. The reason for obtaining a large MR in the AFM phase than in FM phase could be related to the exchange splitting and the canted spin orientation which is more enhanced in the AFM phase than in the FM phase (Fig. 3(b,c)). Similar behavior in the magnetoresistance has been observed for PrGa, where a large magnetoresistance of 34% is reported at 5 T field in AFM phase^41^. Also it has been observed that the MR decreases and becomes more positive in the FM phase in PrGa which is attributed to the change in the lattice parameters during AFM to FM phase transition on the application of field^41^. Another interesting result in PrGe is that an anisotropic MR behavior *w*.*r*.*t*. field direction (Fig. 4(b)) is observed at all the temperatures only for the [010] crystallographic orientation (supplementary information Fig. 4). Similar anisotropic behavior in MR has been observed in the Pt/Co/Pt films^42^, CrO2 thin films^43^ and MnSi nanowires^44^ where it is related to the chiral magnetic structure present in these systems. The chiral magnetic structure lacks inversion symmetry and has a strong spin-orbit coupling which is mainly induced by the DM interaction. It is well known that the origin of the chiral magnetic states is related to the change of lattice structure at the crystal boundary or due to the surface contribution which generates an additional magnetic anisotropy.

DM interaction is expected to influence the lattice structure, hence we have performed temperature dependent x-ray diffraction (XRD) studies on the powder sample to probe its existence. The XRD data have been recorded at few temperatures using laboratory based (Cu K_*α*_) source (Fig. 4(c)) and linearly polarized synchrotron source (13 KeV energy) (Fig. 4(d)). XRD patterns from lab source did not show any change in the peak profiles till the lowest temperature. This indicates no structural change across the magnetic transition and the sample retains CrB type structure with orthorhombic space group Cmcm (inset of Fig. 4(c)).

XRD patterns from the polarized synchrotron source showed some additional peaks as compared to those recorded from lab source. In Fig. 4(d) the strong peak (around 18.7°) shows signature of splitting into two peaks at 18.64° and 18.71° (shown by dotted lines in the Fig. 4(d)) observed in both heating and cooling cycles. On plotting the intensity of the 18.97° peak (peak A in Fig. 4(d)) as a function of temperature in Fig. 4(e), we find a hysteresis around the magnetic transition. Similar kind of hysteresis as a function of temperature has been observed for other peaks, which indicates the first order nature of magnetic transition that is in good agreement with our earlier work on PrGe^19^. DM interaction has been reported to show first order magnetic transition in MnSi^45^. Hence, the magnetic transition in this system are due to strong coupling between the spins and the lattice which further confirms the presence of DM interaction in this system.

The conclusions drawn from the present work are: 1) PrGe behaves like a weak ferromagnetic system in which the Pr atoms in different sublattices have antiparallel spin orientation and the evidence of strong coupling between the spins and the lattice has been obtained in the experiments. 2) Origin of giant Rashba effect on the PrGe [010] surface in the PM phase is due to the breaking of space inversion symmetry by the DM interaction. 3) DM interaction arises in this system due to the hybridization between the magnetic Pr 4*f* electrons with the Pr 5*d* conduction electrons. 4) Magnetic ordering both ferromagnetic and antiferromagnetic arises due to the competition between the DM interaction and the exchange interaction present in this system which actually causes the change in the spin-polarization of the conduction electrons. 5) In the FM and AFM phases, the effective mass of the electrons increases and the change in the spin-polarization causes a finite interaction between the hole-like and the electron-like states. Hence, we propose that the PrGe [010] surface which shows a giant Rashba effect (*α*_*R*_ = 5 eV*Å*) in the PM phase, giant negative MR (43%) in the AFM phase and the possibility to tune the Rashba parameter by varying temperature across the magnetic transitions makes this material a very promising candidate for the AFM spintronics application.

## Methods

### Sample preparation and characterization

PrGe single crystal was grown by the Czochralski method^19^. The sample has been characterized by XRD, magnetization, resistivity, susceptibility and specific heat measurements.

### Angle resolved photoemission

High resolution angle resolved photoemission measurements at 10 meV energy resolution and 0.2 deg angular resolution were performed at the APE beamline of Synchrotron Elettra, Italy^46^. The clean surface of the PrGe single crystal was obtained by cleaving the sample *in*-*situ* in a base pressure of 9 × 10^−11^ mbar. The data have been recorded with a Scienta SES 2002 electron energy analyzer. The temperature dependent data are collected while heating using a liquid helium cooled cryostat. We have performed different data processing on the raw ARPES data (Fig. 1 of supplementary information) and the data presented here are processed using Curvature method^47^.

### Resonant photoemission

The resonant photoemission measurements  were carried out at the angle-integrated PES beamline on the Indus-1 synchrotron radiation source, India^48^. Experimental conditions are similar as reported in other references^27, 48, 49^. The sample surface was scraped *in*-*situ* using a diamond file multiple times to obtain atomically clean surface. The BE in the photoemission spectra has been determined with reference to the Fermi level of the clean gold surface that is in electrical contact with the sample at the same experimental conditions^27^. The intensities of the photoemission spectra were normalized to the photon flux estimated from the photo current of the post mirror at the beamline.

### Density Functional Theory

We have performed the plane-wave based first-principles calculations within the density functional theory (DFT) using the generalized gradient approximation (GGA) for exchange and correlations potential using the parametrization scheme of J. P. Perdew, K. Burke and M. Enzerhof (PBE)^50^. We have used the Vienna ab-initio simulation package (VASP)^51^, which solves the Kohn-Sham equations using a plane wave expansion for the valence electron density and wave functions. The interactions between the ions and electrons are described by the projector augmented wave (PAW)^52^ potentials. For our calculations, we have used the PAW potential for Ge which treats 3*d*4*s*4*p* states as valence and for Pr 5*d*4*f*6*s* (trivalent) states were treated as valence. We have performed the spin-polarized calculations for both bulk and surface structures. The expansion of electronic wave functions in plane waves was set to a kinetic energy cut-off (*E*_*cutoff*_) of 350 eV for both the structures. The Brillouin-zone was sampled using Monkhorst-Pack k-point mesh^53^ of 8 × 8 × 8 (128 k-points in the irreducible Brillouin zone (IBZ)) and 4 × 8 × 1 (16 k-points in the IBZ) for the bulk and surface structure, respectively. Each structure, optimization was carried out with respect to a k-point mesh and *E*_*cutoff*_ to ensure convergence of the total energy within a precision better than 1 meV/atom. The structural relaxations were performed using the conjugate gradient algorithm until the residual forces on the atom were less than 0.01 eV/*Å* and stresses in the equilibrium geometry were less than 5 × 10^−2^ GPa. The total electronic energy and density of states (DOS) calculations were performed using the tetrahedron method with Bl*ö*chl corrections^52^. For calculations of spin- and site-projected DOS for Pr and Ge atoms, the Wigner-Seitz radii chosen were 2.003 *Å* and 1.541 *Å*, respectively.

### Temperature dependent XRD

Low temperature XRD measurements were performed on the angle-dispersive x-ray diffraction beamline on the Indus-2 synchrotron radiation source. A high spectral resolution of about 1 eV at 10 KeV was achieved using Si(111) based double crystal monochromator^54^. Powder XRD of PrGe single crystal was recorded at 13 KeV excitation energy by using Image plate Mar-345 detector. The photon energy and the sample to detector distance were accurately calibrated using LaB6 NIST standard. Fit2D software was used to generate the XRD pattern from the diffraction rings obtained by Image plate data. Low-temperature XRD measurements were also done on x-ray powder diffractometer using rotating anode type x-ray source (Cu K_*α*_) and CCR cryostat.

### Magnetoresistance

The magnetoresistance was measured along the crystallographic [010] direction while the field was applied along the easy axis of magnetization, i.e. the [001] crystallographic direction. The measurements were performed using a Quantum Design built Physical Property Measurement System (PPMS), where the resistivity is measured by means of standard four probe method.

## Electronic supplementary material

Supplementary Information
