# Supplementary material for: Giant Rashba effect at the topological surface of PrGe revealing antiferromagnetic spintronics
Source: Sci Rep. 2017 Jun 23;7:4120. doi: 10.1038/s41598-017-02401-z (PMC5482886; doi:10.1038/s41598-017-02401-z)
Supplement: Supplementary file 1 — Supplementary Information [file 41598_2017_2401_MOESM1_ESM.pdf]

Giant Rashba effect at the topological surface of PrGe revealing antiferromagnetic spintronics

Soma Banik[1,*], Pranab Kumar Das[2], Azzedine Bendounan[3], Ivana Vobornik[4], A. Arya[5], Nathan Beaulieu[3], Jun Fujii[4], A. Thamizhavel[2], P. U. Sastry[6], A. K. Sinha[1], D. M. Phase[7], and S. K. Deb[1]

[1]Synchrotrons Utilization Section, Raja Ramanna Centre for Advanced Technology, Indore, 452013, India.
[2]Department of Condensed Matter Physics and Materials Science, Tata Institute of Fundamental Research, Homi Bhabha Road, Colaba, Mumbai 400005, India.
[3]Synchrotron SOLEIL, L'Orme des Merisiers, Saint-Aubin, BP 48, FR-91192 Gif-sur-Yvette Cedex, France.
[4]Istituto Officina dei Materiali (IOM)-CNR, Laboratorio TASC, in Area Science Park, S.S.14, Km 163.5, I-34149 Trieste, Italy.
[5]Materials Science Division, Bhabha Atomic Research Centre, Mumbai 400085, India.
[6]Solid State Physics Division, Bhabha Atomic Research Centre, Mumbai 400085, India.
[7]UGC-DAE Consortium for Scientific Research, Khandwa Road, Indore 452001, India.
*soma@rrcat.gov.in

Supplementary Information

Supplementary information of this article includes :

1) ARPES Data Processing.

2) Analysis of Energy Dispersion Curves.

3) Magnetoresistance Vs Field as a function of temperature.

Supplementary Information

1) <u>ARPES Data Processing:</u> We have performed the data processing using different methods like 1) the second derivative of the ARPES data and 2) Curvature method (Ref: Z. Peng et al., in Rev. Sci. Instrum. **82**, 043712 (2011)). Both the data processing is shown in Fig. 1. Left panel in Fig. 1 shows the raw data, the middle panel shows the data processing using second derivative and the right panel shows the data processing using the curvature method. We find that the Curvature method is far better than the second derivative method and detail information about the band structure can be obtained from this method.

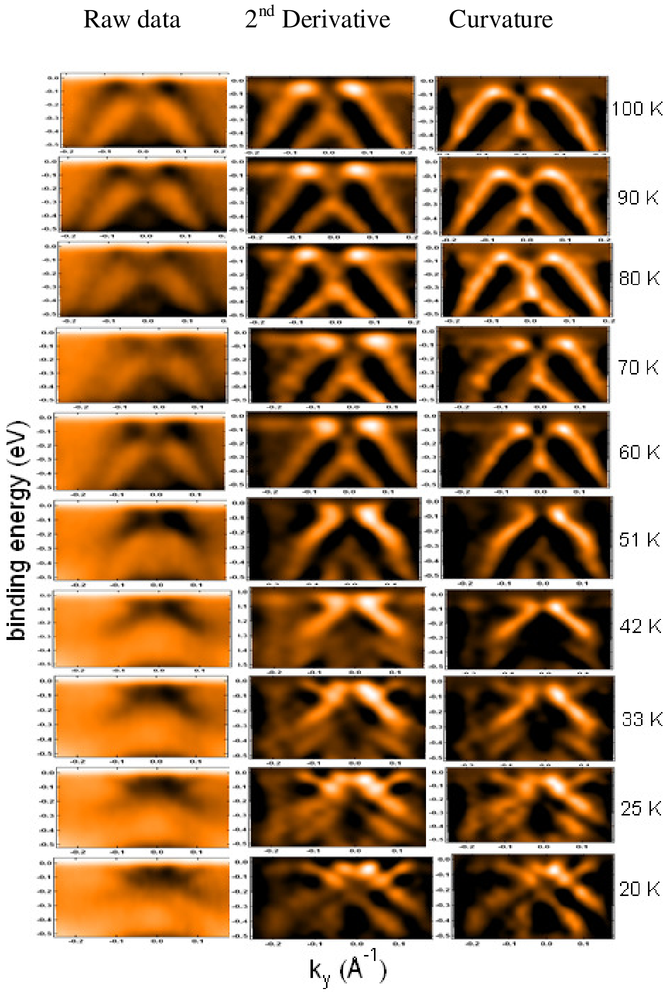

Fig. 1: *Temperature dependent ARPES data, left panel show the raw data, middle panel shows the data processing by taking second derivative and right panel shows the data processing by using curvature method.*

2) Analysis of Energy Dispersion Curves:

The motivation to perform the analysis is to understand the changes in the band structure as observed in the experiment across the magnetic phase transition. Single particle energy for a magnetic system, is described as:

$$E_{k,\sigma} = \hbar^2/2m^*[(k_x - \sigma.k_0 \sin\theta)^2 + k_y^2] - E_R \sin^2\theta - \sigma[(J_0S)^2 + \alpha_R^2 (k_x^2 \cos^2 \theta + k_y^2)]^{1/2} \quad \text{---------------} \ (1)$$

Here, m^* is the effective mass of the electrons, σ is the carrier spin index $\sigma = \pm 1$, θ denotes the angle of the magnetization with respect to the Z-direction, J_0S (denoted as Δ in the text) represents the coupling between the magnetic impurity and the carriers where J_0 is the exchange strength and S is the spin of the magnetic impurity. E_R is the Rashba energy and α_R is the rashba parameter. k_0 is the shift of the bands in the momentum space away from the Γ point. α_R and E_R are function of k_0 and m^* as: $\alpha_R = \hbar^2 k_0/m^*$ and $E_R = m^* \alpha_R^2/2 \hbar^2 = \hbar^2 k_0^2/2m^*$. So any changes in the shape of the bands mainly depend on value of k_0 and m^*.

There can be three possible cases of the magnetization direction in the sample:

A) Magnetization axis may be exactly perpendicular to the surface in that case $\theta=0$ as shown in Fig. 2(a).

B) Magnetization axis is tilted with respect to the Z direction such that $\theta = 45^o$ along a spin quantization axis in the XY plane as shown in Fig. 2(b).

C) The magnetization direction parallel to the Y axis, so that $\theta = 90^o$ as shown in Fig. 2(c). In this case the exchange field will be along the Y-axis couples with the kinetic energy of the electrons which causes the Fermi sea to shift along the X-axis [Ref: Barnes et al., Scientific Reports 4, 4105 (2014)].

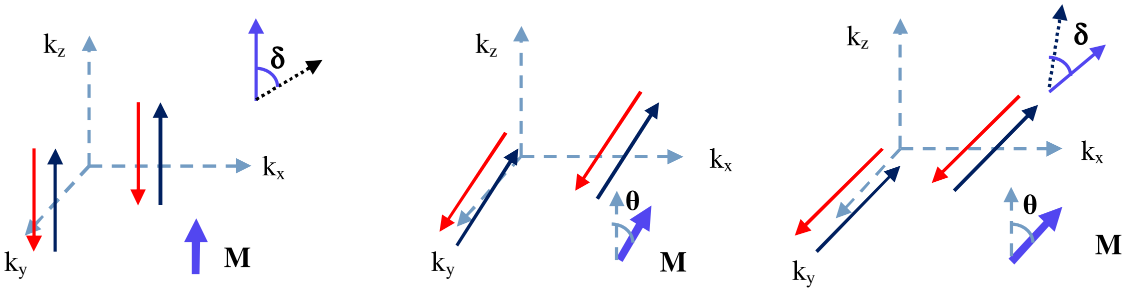

(a) M || Z axis, $\theta = 0^o$ (b) M along Z axis, $\theta = 45^o$ (c) M || Y, $\theta = 90^o$

Fig. 2: *Spin orientations for three different conditions as discussed in the text are shown schematically in (a), (b) and (c). δ is angle of spin canting and the dotted arrow shows the canted spin w.r.t. the magnetization axis (discussed in detail in the text).*

Below in Table 1 and in Fig.3 we have discussed different cases where the effect of k_0, m*, Δ and θ on the band structure are shown. In the experimental band structure (Fig. 1 (c) to (l) of the article) visually one can see that the shift of the bands in the momentum space away from the Γ point is almost constant across the phase transition. Hence, in the stimulation we have kept the value of k_0 (≈0.1) fixed and varied the value of m*, Δ and θ in equation (1) to understand their actual effect on the band structure. Since the value of E_R and α_R depends on k_0 and m*. So for the fixed value of k_0, the change in the shape of the bands and hence the Rashba parameters depends mainly on m*. The value of E_R and α_R are found to decrease for higher effective mass of electrons. m* is considered to be negative and positive to generate hole-like and electron-like bands respectively as observed in the experiment.

Table 1: *Parameters characterizing the spin splitting: wave number offset k_0, effective mass m*, exchange parameter Δ, Angle of magnetization w.r.t Z axis θ, Rashba parameter α_R and Rashba energy of split states E_R.*

S.No.	k_0	m*	Δ	θ	α_R	E_R
a)	0.1	0.15 m_e	0	0	5.34	0.267
b)	0.1	0.55 m_e	0	0	1.45	0.072
c)	0.1	0.15 m_e	0.1	0	5.34	0.267
d)	0.1	0.15 m_e	0	45	5.34	0.267
e)	0.1	0.55 m_e	0	45	1.45	0.072
f)	0.1	0.15 m_e	0.1	45	5.34	0.267
g)	0.1	0.15 m_e	0	90	5.34	0.267
h)	0.1	0.55 m_e	0	90	1.45	0.072
i)	0.1	0.15 m_e	0.1	90	5.34	0.267

Considering the first case (A) when magnetization axis is exactly perpendicular to the surface so that θ=0 (conditions (a) to (c) as listed in the Table 1), the energy dispersive curves are generated in Fig. 3 (a) to (c). We find that the band structure along both the k_x and k_y direction shows the same trend (Fig. 3 (a) and (b)). We can see that the features near the E_F are more enhanced when effective mass is low. The contribution of exchange coupling parameter generates gap between the spin-up (E+) and spin-down bands (E-) near E_F as shown in Fig. 3 (c).

For the second case (B) when magnetization axis is tilted with respect to the Z direction such that θ= 45° (conditions (d) to (f) listed in Table 1), the energy dispersion curves are generated in Fig. 3 (d) to (f). It is quite evident in Fig. 3(d) that when m* is less the bands lie below E_F in the k_y direction but as the m*

increases the spin-up band lie at the E_F as shown in Fig. 3(e). Δ generates a gap below E_F as shown in Fig. 3 (f) in the k_y direction. Asymmetric bands are observed in the k_x direction as shown in Fig. 3 (d) to (f).

For the third case (C) when magnetization direction parallel to the Y axis, so that $\theta = 90^o$ (conditions (g) to (i) listed in Table 1) the energy dispersion curves are shown in Fig. 3 (g) to (i). As it is already discussed that when the magnetization axis is parallel to Y-axis, the Fermi sea will shift along the X-axis and hence can be represented by electron-like band in the k_x direction. It is quite prominent in fig. 3(g) that a gap exists below E_F in the k_y direction but in the k_x direction the electron bands are shifted lowered which depends on the value of E_R. When the m* is higher the gap below E_F reduces and the bands are shifted more towards E_F as shown in Fig. 3(h). Δ generates an additional gap between the spin-up and spin-down bands in the k_y direction. In the k_x direction the Δ generates asymmetric curves as shown in Fig. 3(i).

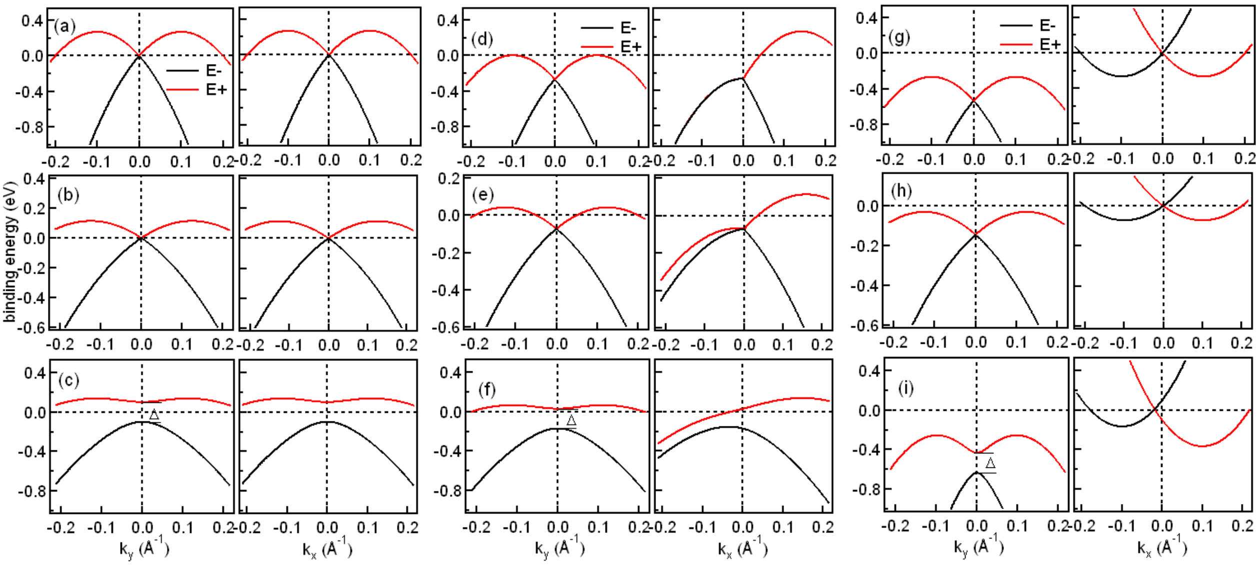

Fig.3: *Energy dispersion curves in k_x and k_y direction for different conditions as listed in Table 1. (a) to (c) shows the energy dispersion curves for the magnetization axis exactly perpendicular to the surface (with respect to Z-axis, $\theta=0$). (d) to (f) shows the energy dispersion curves for the magnetization axis tilted with respect to the Z-direction such that $\theta=45^o$ projected along XY plane. (g) to (i) shows energy dispersion curves for the magnetization axis parallel to the Y axis such that $\theta=90^o$.*

We find that the experimental energy bands as observed in Fig. 1 and Fig. 3 of the present article agrees well with the third condition where we find that the magnetization direction is parallel to the Y-axis and the direction of the moment tilts away from the Y-axis in the direction perpendicular to the surface with the canting angle $\delta(k_y) = \tan^{-1}(\alpha_R.k_y/J_0S)$, as shown in Fig. 2 (c).

3) <u>Magnetoresistance Vs Field as a function of temperature</u>:

In Fig. 4(a) and (b) we have shown the magnetoresistance Vs field as a function of temperature along the [010] and [100] direction respectively. We find a clear anisotropy in the magnetoresistance as a function of positive and negative fields for crystal orientation [010] but this anisotropy is absent for the crystal orientation [100]. This experimental result confirms that the anisotropy observed for the [010] single crystal is real and not the artifact.

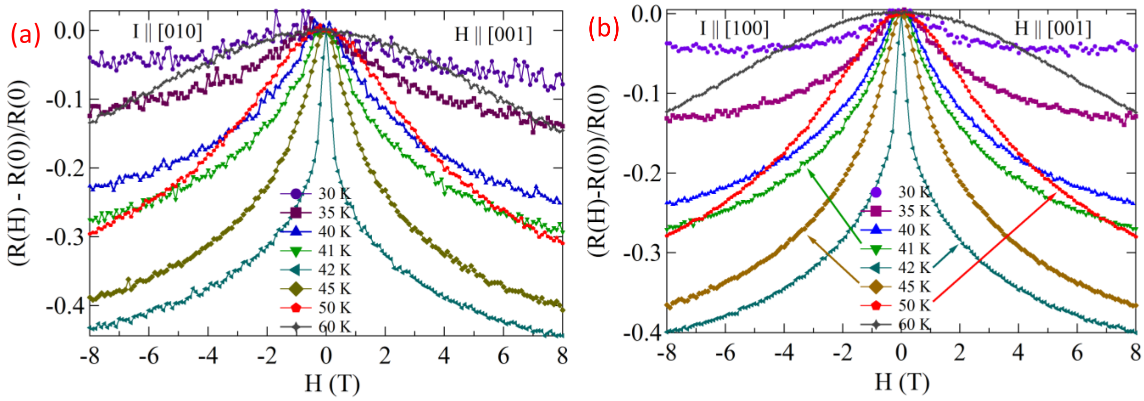

Fig. 4: *MR Vs H as a function of temperature for PrGe at (a) [010] and (b) [100] crystallographic orientations.*
